# A Preliminary Psychometric Study of the Sub-Dimensions of the SCOOHPI Scale for Schizophrenia Patients

**DOI:** 10.3390/bs16060938

**Published:** 2026-06-08

**Authors:** Mohamad Hamad, Nathalie Rude, Ibrahim Y. Tawbe, Moustafa Fayad, Francesca Siu-Paredes, Frédéric Denis

**Affiliations:** 1 Faculty of Science, Lebanese University, Beirut 1107, Lebanon; 2 Research Laboratory “Intégratives en Neurosciences et Psychologie Cognitive”, LINC, Faculty of Health, Université Marie et Louis Pasteur, 25000 Besançon, France; 3 Research Laboratory “Aménagement des Usages des Ressources et des Espaces Marins et Littoraux”, University of Bretagne Occidentale, 29000 Quimper, France; 4 SINERGIES (UR 4662), Université Marie et Louis Pasteur, 25030 Besançon, France; 5 Dentistry Faculty, Université de Reims Champagne Ardenne, 51100 Reims, France; 6 EA 75-05 Éducation Éthique Santé, Faculty of Medicine, François-Rabelais University of Tours, 37044 Tours, France

**Keywords:** psychometric validation, SCOOHPI, structure validity, sub-dimensions, principal component analysis, coping strategies

## Abstract

Background: The SCOOHPI scale (Schizophrenia Coping Oral Health Profile and Index) assesses the coping strategies of schizophrenic patients in terms of oral health. In this article, we explore the sub-concepts assessed by the SCOOHPI scale to use it as a profile. Methods: We studied the internal consistency of the items of the SCOOHPI scale; then, the structural validity of this scale was examined using a principal component analysis. Finally, we studied the relationship between the SCOOHPI scale and sociodemographic and clinical variables. Results: The total sample consisted of 96 participants, including 72% men and 59% smokers. The SCOOHPI scale shows good internal consistency (α = 0.84). The principal component analysis reveals the existence of three sub-concepts assessed by the SCOOHPI scale. Conclusion: This study made it possible to examine the sub-dimensions of the SCOOHPI scale. This tool can contribute to a better assessment of the coping strategies of schizophrenic patients in terms of oral health. This study provides a preliminary validation of the SCOOHPI scale as a profile. Further confirmatory studies on independent samples are needed to confirm its factor structure.

## 1. Introduction

Measuring subjectivity in healthcare is essential to help clinicians better understand their patients and support them in their treatment choices. It is therefore crucial to have reliable and validated tools, particularly in contexts where subjectivity has a significant impact, such as in individuals with schizophrenia. Indeed, in the context of disorders associated with schizophrenia, numerous mental disturbances can alter thinking, such as delusions or hallucinations. In this context, it becomes particularly difficult to perceive what the patient is truly thinking and, consequently, to guide them effectively to improve their physical and mental health.

The “Schizophrenia Coping Oral Health Profile and Index” (SCOOHPI) scale was developed to document the coping strategies of individuals with schizophrenia in the face of oral health problems. The study of coping strategies in schizophrenia has received growing attention, particularly in first-episode psychosis (Riera-López de Aguileta et al., 2020). This 18-item scale has psychometric properties that ensure its unidimensionality (Hamad et al., 2022), allowing for the generation of a score reflecting the respondents’ level of coping. However, an overall score cannot capture the complexity and diversity of coping strategies. It was therefore relevant to pursue the psychometric analysis of this scale to determine whether it could also be used as an index, or whether subscores could reflect the different facets of strategies adopted.

The SCOOHPI scale assesses coping strategies related to the specific oral health of patients with schizophrenia (Siu-Paredes et al., 2021), and schizophrenia affects 1% of the general population (Jablensky, 2000). Studies concerning this mental illness are therefore crucial, hence the importance of studying the complete psychometric validation of the subjective measures used, particularly the SCOOHPI scale. This scale aims to help healthcare professionals improve the ability of patients with schizophrenia to cope with their oral health. Coping, or adaptation strategies, constitutes the thoughts and behaviors used by patients to deal with stressful or difficult situations (Folkman & Moskowitz, 2004). Adaptation is a person’s ability to react to a stressor; coping strategies are specific to each individual (Siu-Paredes et al., 2021). People with schizophrenia tend to have pervasive cognitive problems, personality alterations, self-esteem issues, and social dysfunctions. All these deficiencies reduce the effectiveness of learning new strategies to improve their general health and particularly their oral health (Chu et al., 2012; Paredes et al., 2019). In this context, understanding the reactions of an individual suffering from schizophrenia and oral disorders is essential in order to best support these individuals.

The psychometric validation of subjective measurement scales is a fundamental procedure for the use of any measurement scale. This procedure consists of several steps that must be carried out in order.

The first step of psychometric validation begins during the scale development with content validity, where it is shown that the scale assesses a set of characteristic attributes (Lynn, 1986). Next, perceived validity verifies that the observed individuals understand the questionnaire; this is also referred to as an acceptability study (Mosier, 1947).

The second step of psychometric validation studies the reliability of the scale. In other words, it is shown that the items are consistent with the scale’s concept (Cronbach, 1951); then, reliability over time is studied by conducting a test–retest (Bland & Altman, 1986), with the aim of ensuring that individuals do not answer the questionnaire arbitrarily. To complete this step, we study the scale’s sensitivity to change (Fermanian, 2005), for example, after the introduction of a treatment or a prevention campaign.

The last step of psychometric validation is the stage during which the scale’s score is explained. First, construct validity shows that the scale measures the concept it is supposed to measure, by comparing this scale to other scales that measure similar concepts (Campbell & Fiske, 1959). Finally, we study the structure of the scale, meaning we show whether the scale is unidimensional (or is an index) and/or if there are multiple sub-dimensions (in this case, we speak of a profile) (Mokkink et al., 2010). An ideal scale can be both an index and a profile.

Initially, when developing a scale, we may be led to make hypotheses about the structure of the scale (on the grouping of certain items); it is during the structural validity step that these hypotheses are verified or disproven.

The overall concept studied, here coping, can be broken down into several sub-concepts (or facets). In this case, the scale may include subscales or groups of items that measure these facets or sub-concepts. This is why, in this article, we study the structure of the SCOOHPI scale using a principal component analysis (PCA) (Hotelling, 1933).

The aim of this study is to conduct a preliminary psychometric evaluation of the SCOOHPI scale as a profile, using exploratory principal component analysis.

## 2. Methodology

### 2.1. SCOOHPI Scale Items

The SCOOHPI scale consists of 18 items assessing coping strategies (or coping), of which 4 items (items 5, 9, 15, and 18) are inversely recoded to make them positive regarding strategies promoting the improvement of patients’ daily lives. Each item has 5 response modalities (Never, Rarely, Sometimes, Often, and Always). The items of the SCOOHPI scale allow for the exploration of 3 dimensions: physical well-being strategies, moral well-being strategies, and access to oral health well-being strategies (Siu-Paredes et al., 2021).

Less than 5% of the data are missing. Thus, we use a simple imputation method, and missing values are replaced by the mean (Downey & King, 1998).

All analyses were performed using R software version 4.4.1.

### 2.2. Variables Studied

The variables collected in this study were gender, height, weight, smoking status, number of antipsychotics, TTC (time to care), and two oral health indices: the DMFT index (total of Decayed, Missing, and Filled Teeth) and the OHI-S index (Simplified Oral Hygiene Index).

### 2.3. Study Population and Data Collection

Our study is conducted on a sample of 96 schizophrenic patients from 5 hospital centers: the Chartreuse center (13 patients), the Châlons center (8 patients), the Tours center (18 patients), the Millau center (34 patients), and the Paris center (23 patients).

Patients were referred to the study investigator by the healthcare professional responsible for their mental healthcare. They were informed by the investigator about the nature, objectives, methodology, duration, expected benefits, constraints, and foreseeable risks of the research, in accordance with Article L1122-1 of the French Public Health Code.

Participants were informed that their data would be computerized, kept confidential, and processed anonymously and that they could access and rectify their data at any time.

### 2.4. Structural Validity

Structural validity studies whether the concept of our scale is explored by an overall score and whether this scale also explores sub-concepts. In other words, we study whether our scale is unidimensional and whether sub-dimensions exist (Mokkink et al., 2010).

Several methods are used to study structural validity. In this article, the structure of the SCOOHPI scale is studied using principal component analysis (PCA).

### 2.5. Principal Component Analysis

#### 2.5.1. Objectives

The main objective of principal component analysis (PCA) is to represent the responses of individuals in a reduced-dimension space while preserving as much information as possible (at least 70% of the cumulative variance must be expressed). PCA is therefore performed if there are at least 3 variables (Hotelling, 1933).

Exploratory factor analysis (EFA) is theoretically more appropriate for modeling the common variance of a reflective construct. In this article, we chose PCA for this exploratory study. Our objective is to describe the total variance of the items in order to empirically identify groupings, from a purely descriptive and non-confirmatory perspective. This approach is still commonly used in the early stages of structural validation of health scales (Schönenberg et al., 2022; Liu et al., 2022; Chan et al., 2025).

Principal component analysis with Varimax rotation has been successfully used to explore the factor structure of coping strategies in schizophrenia (Shah et al., 2017).

#### 2.5.2. Kaiser–Meyer–Olkin Index

The KMO (Kaiser–Meyer–Olkin) index is an index of the adequacy of the factorial solution; items are compatible with a factorial analysis as soon as the KMO value exceeds 0.5 (Kaiser & Rice, 1974). In other words, items should be removed or the scale should be divided into 2 or more sub-dimensions if this index is below 0.5. Some articles in the literature remove items when this index is below 0.6 (Tavakol & Dennick, 2013; Yang-Wallentin et al., 2010).

#### 2.5.3. Bartlett’s Test of Sphericity

Bartlett’s test of sphericity measures whether the correlation matrix diverges from the identity matrix, meaning this test measures the possibility of reducing the number of scale dimensions (Pett et al., 2003). If all variables are perfectly correlated, then a single dimension is sufficient to explain the model. Conversely, if the correlations between variables are zero, then each variable must be represented by one dimension.

The null hypothesis of Bartlett’s test of sphericity is that the correlation matrix equals the identity matrix, and the alternative hypothesis is that the correlation matrix differs from the identity matrix.

#### 2.5.4. PCA Methodology

The total moment of inertia of the cloud of individuals relative to the center of gravity is calculated; a distance must be chosen to calculate this moment (normally, the Euclidean distance is used). This moment of inertia measures the dispersion of the cloud of individuals relative to the center of gravity; the greater the moment of inertia, the more dispersed the individuals are.

When projecting individuals onto a space of smaller dimension than the initial space’s dimension, the total inertia decreases. In this case, a subspace is sought so that as little information as possible is lost during projection. According to the Kaiser rule, dimensions with an eigenvalue greater than 1 are generally retained (Jackson, 1993). However, it is also common to use a 70% cumulative variance threshold as a guide, particularly in an exploratory approach. In our study, we chose to retain the dimensions allowing us to reach 70% cumulative variance (Cattell, 1966; Shaharudin & Ahmad, 2017).

Over the entire observed point cloud, we search for factors that could explain the results. In other words, we investigate whether several dimensions of the studied concept emerge by determining the affiliation of each variable (item in our case) to the studied factors. In practice, the polychoric correlation matrix is calculated, and then axes are identified using eigenvalues (Abdi & Williams, 2010).

#### 2.5.5. Rotation of Factorial Axes

The principal components of PCA are sometimes difficult to interpret, as there can be many variables with significant correlations on several factorial axes. The Varimax rotation of axes maximizes the strongest loadings and minimizes the weakest ones so that each factor that emerges is determined by a single group of variables (Kaiser, 1958). The rotation method makes these correlation values more distinct, thus facilitating the interpretation of the factors. There are several types of rotation; Varimax rotation is the most commonly used.

Varimax rotation was developed by (Kaiser, 1958). This rotation aims to obtain, for each factor, a small number of high scores and a large number of zeros (values close to zero), thereby preserving the orthogonality of the factorial axes. After Varimax rotation, interpretation becomes much simpler: each item should be associated with one factor, and each factor represents a single set of items (or facet). Furthermore, each factor can be interpreted based on the opposition of a few variables according to other factors.

Varimax rotation is applied by maximizing *V*,

$$V\;=\;\sum_{j=1}^{k}\sum_{l=1}^{p}{\left(q_{j,l}^{2}-{\bar{q}}_{l}^{2}\right)}^{2}$$

where k is the number of items, $q_{j,l}^{2}$ is the square of variable j on factor l, and ${\bar{q}}_{l}^{2}$ is the mean of the squared loadings for factor l.

The orthogonal solution is always preferable because it indicates that each factor contributes a unique solution, not shared by another factor.

## 3. Results

### 3.1. Sample Description

Our sample comprises 96 individuals. The percentage of men is 72%, the percentage of smokers is 59%, the average weight of individuals is 79 kg, and the average height of individuals is 172 cm.

### 3.2. Internal Consistency

Cronbach’s alpha is 0.84 (>0.7), which shows that the items of the SCOOHPI scale are consistent with the scale’s concept, coping (Cronbach, 1951).

### 3.3. Principal Component Analysis with Varimax Rotation

The KMO (Kaiser–Meyer–Olkin) index is 0.81 (>0.5); therefore, PCA can be used to study the structure of the SCOOHPI scale.

The items are ordinal in nature (five-point Likert scale); thus, PCA was performed on a polychoric correlation matrix (Holgado–Tello et al., 2010).

The polychoric correlation matrix between the items shows the existence of a good correlation between certain items (>0.3) and the absence of correlation between other items (<0.1). Thus, a PCA reduces the number of dimensions of the SCOOHPI scale and shows the sub-concepts of this scale (Appendix D).

Bartlett’s test of sphericity shows that the correlation matrix is significantly different from the identity matrix (*p*-value = 3.90 × 10^−75^ < 0.05). PCA can therefore be performed on the SCOOHPI scale in order to determine its dimensions.

According to Kline’s guidelines (Kline, 2011), absolute skewness values greater than 3 and absolute kurtosis values greater than 8 to 10 indicate severe non-normality. Our results (skewness between −1.12 and 0.23; kurtosis between −1.48 and −0.02) fall well within these limits, confirming that the data do not severely deviate from normality (Table 1).

Bartlett’s test of sphericity shows that the correlation matrix is significantly different from the identity matrix (*p*-value = 3.90 × 10^−75^ < 0.05). PCA can therefore be performed on the SCOOHPI scale in order to determine its dimensions.

The PCA is performed with Varimax rotation on the 18 items. A single factor (comp 1 in Table 1) represents 32% of the information (insufficient), and six factors represent 71% of the information (Table 1).

The PCA is performed with Varimax rotation on the 18 items. A single factor (comp 1 in Table 2) represents 32% of the information (insufficient), and six factors represent 71% of the information (Table 2).

The Varimax and oblimin rotations yielded highly similar factor loadings. The only notable difference concerned item 10, which had a slightly higher cross-loading on factor 3 in the oblimin solution (0.54 vs. 0.53) compared with a single loading on factor 1 in the Varimax solution (0.59). Given the weak factor correlations in the oblimin solution (maximum r = 0.314), the Varimax solution was retained. The complete oblimin results are available in Appendix B.

In accordance with standard psychometric recommendations (Costello & Osborne, 2005), only components with at least three items are considered interpretable dimensions. Components 4 (one item), 5 (one item), and 6 (two items) are not retained as stable dimensions in this study. In addition, Velicer’s MAP test and the Very Simple Structure (VSS) criterion both recommended retaining three components, which supports our focus on the first three dimensions as stable (Revelle & Rocklin, 1979; Velicer, 1976).

**Figure 1:**
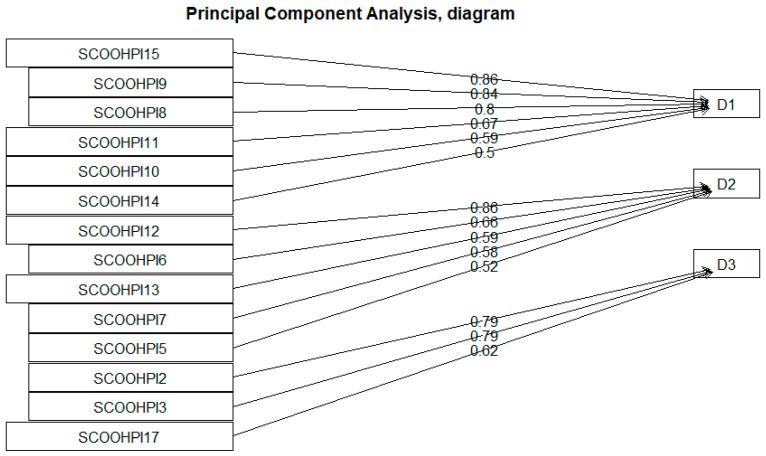
Distribution of items across dimensions.

The analysis reveals three main dimensions, each with at least three items. The first dimension (Oral Hygiene) includes items 8, 9, 10, 11, 14, and 15. The second dimension (Lifestyle Hygiene) includes items 5, 6, 7, 12, and 13. The third dimension (Activities and Organization) includes items 2, 3, and 17. Items 1, 4, 16, and 18 did not form stable dimensions (less than three items per factor) and are not interpreted in this study (Table 3, Figure 1).

**Figure 2:**
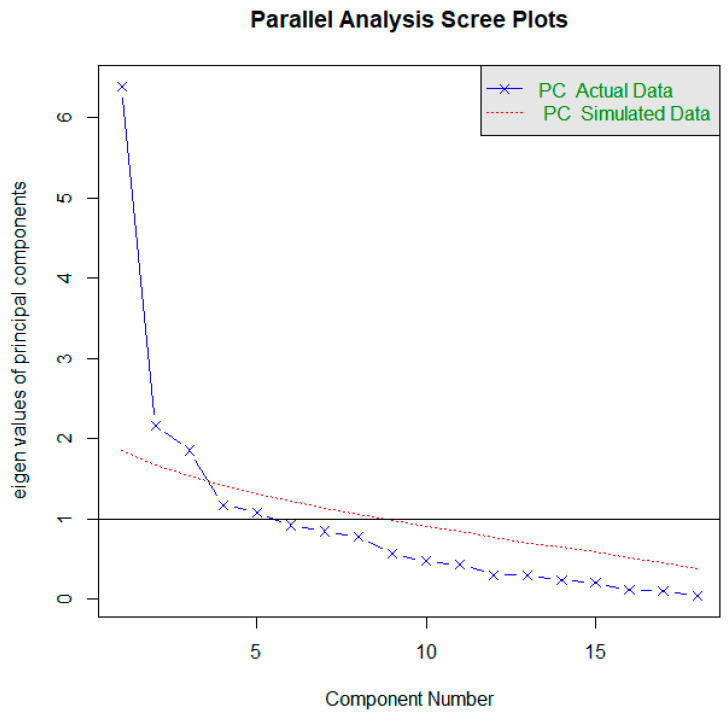
Parallel analysis.

To confirm the number of stable dimensions, parallel analysis (Monte Carlo, 100 permutations) was performed on the polychoric correlation matrix. As shown in Figure 2, the observed eigenvalues exceeded the random eigenvalues for the first three components only. Parallel analysis therefore recommended retaining three components, which supports our focus on the first three dimensions as stable.

Subscale scores were calculated by summing the corresponding items after reverse coding (items 5, 9, 15, and 18).

The full rotated component matrix (Varimax) with all loadings is available in Appendix C.

The internal consistency of the three stable dimensions was assessed using Cronbach’s α and McDonald’s ω (Table 4). Dimension 1 (Oral Hygiene) showed good reliability (α = 0.87, ω = 0.92), Dimension 2 (Lifestyle Hygiene) showed acceptable reliability (α = 0.75, ω = 0.82), and Dimension 3 (Activities and Organization) showed lower but acceptable reliability for an exploratory study (α = 0.62, ω = 0.68). Inter-correlations between the three dimensions ranged from 0.18 to 0.54, and correlations with the total score ranged from 0.50 to 0.89 (Table 5).

The sub-dimensions of the SCOOHPI scale cover the various components of the scale’s concept, i.e., the coping strategies related to the specific oral health of patients with schizophrenia:

The first dimension, Oral Hygiene, assesses behaviors related to tooth brushing, mouth care, and oral hygiene management.

The second dimension, Lifestyle Hygiene, studies general health habits including diet, personal hygiene, and hydration.

The third dimension, Activities and Organization, takes into account the ability to carry out daily activities, to go out, and to organize one’s care.

### 3.4. Relationship Between the Sub-Dimensions of the SCOOHPI Scale and Demographic and Clinical Variables

**Figure 3:**
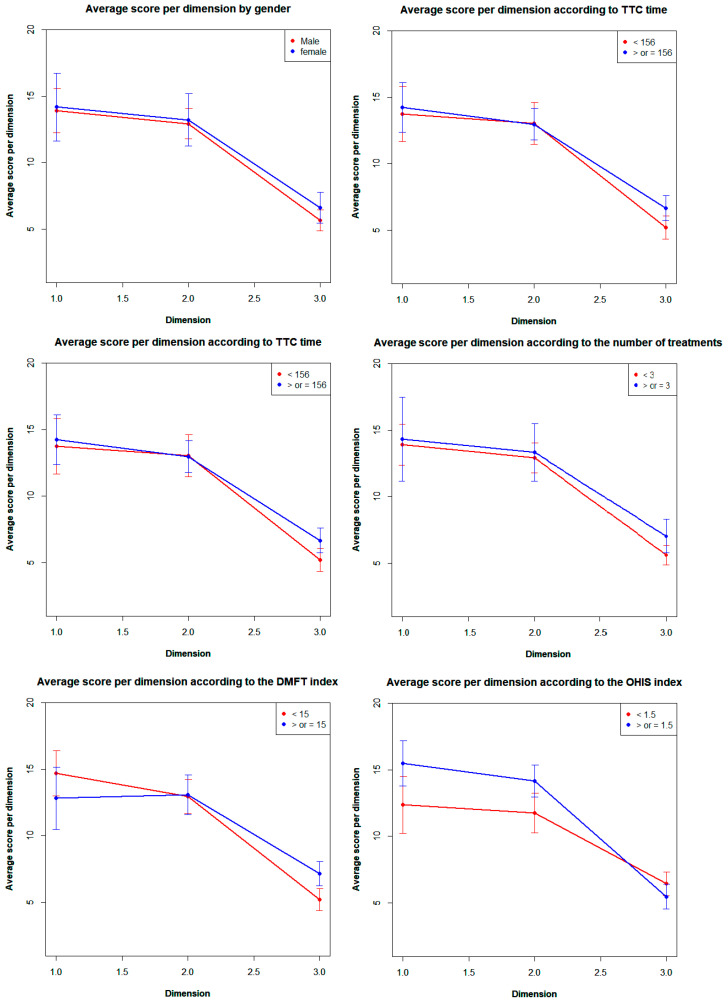
Relationship between the 6 dimensions of SCOOHPI and demographic and clinical variables.

We study the relationship of the dimensions of the SCOOHPI scale with sociodemographic and clinical variables. According to Figure 3, the dimensions of the SCOOHPI scale are not sensitive to these variables, except for the third dimension (Activities and Organization), which is sensitive to the DMFT index (total of Decayed, Missing, and Filled Teeth), with a *p*-value of 0.0035 and a medium effect size (Cohen’s d = −0.63, 95% CI [−1.06, −0.20]). After Bonferroni correction for 18 comparisons (3 dimensions × 6 clinical variables), this association was no longer statistically significant (adjusted threshold *p* < 0.0028). The higher the DMFT index, the higher the mean score of dimension 3, “Activity and Organization.”

The 95% confidence intervals of the means were calculated using the normal approximation (*n* > 30). The absence of overlap of the confidence intervals in Figure 3 indicates a statistically significant difference (*p* < 0.05).

## 4. Discussion

In this article, we study the sub-concepts of the SCOOHPI scale. Using principal component analysis with Varimax rotation, we show that the SCOOHPI scale comprises three sub-scales: “Oral Hygiene”, “Lifestyle Hygiene” and “Activities and Organization”.

These results should be interpreted as preliminary validation, given the exploratory design and modest sample size.

### 4.1. Internal Consistency

A Cronbach’s alpha of 0.84 shows that the items of the SCOOHPI scale are similar in their content, meaning they reflect the coping concept well, and therefore indicates good internal consistency.

### 4.2. Relationship Between the Sub-Dimensions of the SCOOHPI Scale and Demographic and Clinical Variables

According to the study of the relationship between the three dimensions of the SCOOHPI scale and the sociodemographic and clinical variables, we find that only the third dimension (Activity and Organization) showed a nominally significant association with the DMFT index before correction for multiple comparisons (*p* = 0.0035). After Bonferroni correction, this association was no longer statistically significant. Nevertheless, the medium effect size (Cohen’s d = −0.63) suggests that the patient’s dental condition may affect their ability to organize activities and, in particular, their ability to make dental appointments, a finding that merits further investigation in larger samples.

### 4.3. Structural Validity

Factor analysis can be used to study structural validity. The main idea of this method is to reduce the number of factors as much as possible, where initially each variable (item) corresponds to one factor. Thus, this method in practice leads to exploring the sub-dimensions of scales during psychometric validation, which is clearly reflected in our results and in the literature. For example, “the Second Victim Experience and Support Tool (C-SVEST)” (Chen et al., 2019), “questionnaire to measure post-intensive care syndrome” (Jeong & Kang, 2019), “Chinese empathy motivation scale” (Zhu et al., 2019), and “The Stigma of Suicide Scale” (Ludwig et al., 2020) use factor analysis with the aim of obtaining the sub-concepts of their scale.

The SCOOHPI scale assesses coping strategies related to the specific oral health of patients with schizophrenia (Siu-Paredes et al., 2021). The principal component analysis of our scale reveals the existence of three subscales: “Oral Hygiene”, “Lifestyle Hygiene”, and “Activity and Organization”. Each of these dimensions contributes to capturing an essential and specific aspect of oral health coping strategies in patients with schizophrenia. Oral Hygiene assesses direct oral care behaviors, reflecting the patient’s ability to maintain a routine despite the illness. Lifestyle Hygiene informs about general habits affecting oral health, illustrating how the patient adjusts their lifestyle to limit dental risks. Finally, activities and organization evaluate the structuring and planning of care, reflecting the use of cognitive strategies to compensate for executive deficits. Therefore, all these dimensions provide a comprehensive and multidimensional assessment of the SCOOHPI scale’s concept.

We have shown that the SCOOHPI scale comprises three main dimensions; however, the partial credit model also demonstrates the scale’s unidimensionality (Hamad et al., 2022). This is not contradictory but allows the SCOOHPI scale to be both a profile and an index. Thus, one can calculate a score per dimension and an overall score. The advantage of having both a profile and an index facilitates interpretations and comparisons from one group of subjects to another. Indeed, differentiating scores by dimension across subpopulations allows for arguing for the scale’s sensitivity.

### 4.4. Limitations of the Study

Our study nevertheless has certain limitations. First, the male overrepresentation (72%) and the high proportion of smokers (59%) in our sample could limit the applicability of the observed factorial structure to other subgroups of schizophrenic patients (particularly women or non-smokers).

Second, the patient inclusion period occurring during the COVID-19 pandemic lockdown raises the question of whether the observed data were biased for the SCOOHPI scale. It is evident that patients were socially isolated and lacked psychiatric and oral healthcare. Thus, we can assume that the patients’ coping strategies were particularly challenged. For example, the items “I go out of my home,” “I engage in a leisure activity (music, singing, drawing, cinema, walking…),” and “I organize to go to the dentist” were negatively impacted by the lockdown. In particular, the item “I organize to go to the dentist” shows the greatest response difficulty, which may be linked to the pandemic, which caused a disruption in health services.

Third, our study has a limitation concerning the sample size (96 participants). This number corresponds to all schizophrenic patients who agreed to participate in the study across the five participating hospitals. Recruiting this clinical population is particularly difficult, which explains the modest sample size. Although the participant-to-item ratio (5.3:1) falls within the range of common practices in exploratory factor analysis (Costello & Osborne, 2005), a larger sample would have been preferable for greater stability of the results. The results presented should therefore be interpreted with caution.

Fourthly, a methodological limitation of our study is the use of PCA rather than exploratory factor analysis. PCA does not separate common variance from unique variance, which is less rigorous for a reflective construct such as coping. Future studies using EFA or confirmatory factor analysis on an independent sample would be necessary to confirm the observed factor structure.

Finally, bootstrap confidence intervals for the loadings were unstable due to the modest sample size (n = 96), consistent with previous findings that bootstrap estimates can be imprecise in small samples (Nevitt & Hancock, 2001). Therefore, bootstrap results are not reported.

## 5. Conclusions

The purpose of studying the structural validity of a scale is to explore the scale’s unidimensionality and to examine its ability to investigate other sub-concepts. If this unidimensionality is demonstrated, we then obtain an index allowing for the establishment of a single score for all items of the scale, which facilitates an overall view of the studied concept. Furthermore, if the scale investigates sub-concepts, this allows for the establishment of multiple groupings of items, resulting in sub-scores for each of the subscales and thus constituting a profile. Ideally, it therefore seems useful to achieve a scale that is both an index and a profile.

Finally, we hope that the SCOOHPI scale will contribute to improving the assessment of oral health in individuals with schizophrenia. Pending confirmatory validation, the SCOOHPI scale appears usable as both a profile and an index in clinical and research settings.

## Figures and Tables

**Table 1:** Skewness and kurtosis of the 18 items of SCOOHPI.

Item	Skewness	Kurtosis
SCOOHPI 1	−0.78	−0.33
SCOOHPI 2	−0.26	−1.09
SCOOHPI 3	−0.23	−1.07
SCOOHPI 4	−0.46	−0.87
SCOOHPI 5	−0.52	−1.24
SCOOHPI 6	−0.44	−0.74
SCOOHPI 7	−1.12	−0.02
SCOOHPI 8	−0.34	−1.26
SCOOHPI 9	−0.16	−1.41
SCOOHPI 10	−0.25	−1.17
SCOOHPI 11	−0.18	−1.37
SCOOHPI 12	−0.62	−0.55
SCOOHPI 13	−0.52	−1.16
SCOOHPI 14	−0.90	−0.88
SCOOHPI 15	−0.09	−1.12
SCOOHPI 16	−0.37	−1.48
SCOOHPI 17	0.23	−1.45
SCOOHPI 18	−0.72	−0.92

**Table 2:** PCA table showing all factors.

Factor	Eigenvalue	Percentage of Variance	Cumulative Percentage of Variance
comp 1	5.7091639	31.7175772	31.71758
comp 2	2.0594185	11.4412138	43.15879
comp 3	1.7572331	9.7624060	52.92120
comp 4	1.1926523	6.6258462	59.54704
comp 5	1.0735598	5.9642212	65.51126
comp 6	0.9186840	5.1038000	70.61506
comp 7	0.8362917	4.6460649	75.26113
comp 8	0.8118744	4.5104136	79.77154
comp 9	0.6420064	3.5667023	83.33825
comp 10	0.5202763	2.8904239	86.22867
comp 11	0.4806060	2.6700331	88.89870
comp 12	0.4082228	2.2679043	91.16661
comp 13	0.3942605	2.1903363	93.35694
comp 14	0.3240468	1.8002599	95.15720
comp 15	0.3093685	1.7187136	96.87592
comp 16	0.2177479	1.2097104	98.08563
comp 17	0.2004225	1.1134583	99.19908
comp 18	0.1441647	0.8009151	100.00000

Note: Items 5, 9, 15, and 18 were reversed (reverse-coded) before analysis.

**Table 3:** Item coordinates.

Items	Factor 1	Factor 2	Factor 3	Factor 6	Factor 4	Factor 5
SCOOHPI 8	0.80					
SCOOHPI 9	0.84					
SCOOHPI 10	0.59					
SCOOHPI 11	0.67					
SCOOHPI 14	0.50					
SCOOHPI 15	0.86					
SCOOHPI 5		0.52				
SCOOHPI 6		0.66				
SCOOHPI 7		0.58				
SCOOHPI 12		0.86				
SCOOHPI 13		0.59				
SCOOHPI 2			0.79			
SCOOHPI 3			0.79			
SCOOHPI 17			0.62			
SCOOHPI 1				0.56		
SCOOHPI 4				0.76		
SCOOHPI 18					0.87	
SCOOHPI 16						0.91

**Table 4:** Reliability of the three stable dimensions.

Dimension	Number of Items	Cronbach’s α	McDonald’s ω
1. Oral Hygiene	6	0.87	0.92
2. Lifestyle Hygiene	5	0.75	0.82
3. Activities and Organization	3	0.62	0.68

**Table 5:** Correlations between the three dimensions and with the total score.

	Dim1 (Oral Hygiene)	Dim2 (Lifestyle Hygiene)	Dim3 (Activities)	Score Total
**Dim1**	1.00	0.54	0.24	0.89
**Dim2**	0.54	1.00	0.18	0.79
**Dim3**	0.24	0.18	1.00	0.50
**Score total**	0.89	0.79	0.50	1.00

## Data Availability

The data presented in this study are not publicly available due to privacy and ethical restrictions, as they involve patient health information from individuals with schizophrenia across multiple hospital centers. No publicly archived dataset was generated or analyzed in this study. Descriptive summary statistics (sample size, sex distribution, smoking status, etc.) are reported within the article.

## Abbreviations

The following abbreviations are used in this manuscript:

SCOOHPI	Schizophrenia Coping Oral Health Profile and Index
PCA	Principal component analysis
KMO	Kaiser–Meyer–Olkin
DMFT	Total of Decayed, Missing, and Filled Teeth
EFA	Exploratory factor analysis

## Appendix A. The SCOOHPI Scale

**Actuellement et/ou au cours des 15 derniers jours, en relation avec ma santé buccodentaire, j’ai pu me dire:**	**Jamais (0)**	**Rarement (1)**	**Parfois (2)**	**Souvent (3)**	**Toujours (4)**
**Je me crée des plaisirs simples (promenade, boire un café, écouter de la musique, regarder la télé…)** ***I am looking for simple pleasures (walk, drink coffee, listen to music, watch TV…)***					
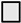
**Je sors de chez moi** ***I go out of my home***					
**Je pratique une activité de loisirs (musique, chant, dessin, cinéma, promenade…)** ***I have a hobby (music, singing, drawing, movie, and ballads …)***					
**Quand je suis dans l’action, je me sens bien** ***When I move, I feel good***					
**Je me sens tenu(e) en otage par le sucre** ***I feel trapped by my relationship with sugar***					
Je mange équilibré ***I have a balanced diet***					
**Je pense à me laver (douche, bain, toilette)** ***I think about washing myself (shower, bath, cleaning)***					
**Je me brosse les dents et/ou mon appareil dentaire** ***I brush my teeth and/or my denture***					
Je néglige mon hygiène buccodentaire ***I neglect my oral health***					
**Je prends soin de ma bouche pour avoir une bonne haleine** ***I take care of my mouth to have a good breath***					
**Je prends soin de ma bouche pour avoir une bonne dentition** ***I take care of my mouth to have a good dentition***					
Je mange sainement ***I eat healthy food***					
**Je pense à boire de l’eau (plate ou pétillante) quand j’ai la bouche sèche** ***I think about drinking water (normal or sparkling) when my mouth is dry***					
**J’arrive à coordonner le mouvement de mes mains pour me brosser les dents** ***I can coordinate the movement of my hands in order to brush my teeth***					
**J’oublie de me brosser les dents** ***I forget to brush my teeth***					
**L’alcool, le tabac, les drogues ont des effets négatifs sur la santé buccodentaire** ***Alcohol, tobacco, drugs have negative effects on the oral health***					
**Je m’organise pour aller chez le dentiste** ***I manage to visit my dentist***					
**J’ai peur d’aller chez le dentiste** ***I’m afraid to go to the dentist***					

## Appendix B. Item Coordinates (Oblimin Rotation)

	**Factor 1**	**Factor 3**	**Factor 2**	**Factor 6**	**Factor 4**	**Factor 5**
SCOOHPI1	0.012889	0.100431	0.311465	0.629999	−0.16145	−0.20764
SCOOHPI2	0.080682	−0.0066	0.740801	0.210973	0.019254	−0.13754
SCOOHPI3	−0.08428	0.036257	0.773481	0.160872	−0.26312	−0.01528
SCOOHPI4	0.167857	0.011196	0.016379	0.755191	0.281144	0.188389
SCOOHPI5	0.098232	0.623274	−0.19028	−0.25086	0.097153	0.09716
SCOOHPI6	−0.26712	0.664212	0.086036	0.290629	0.241394	−0.02092
SCOOHPI7	0.376129	0.521363	0.025905	0.387877	−0.05648	0.042031
SCOOHPI8	0.783583	0.203499	0.166406	0.089271	−0.04401	−0.02044
SCOOHPI9	0.911357	−0.14814	−0.02786	0.058262	0.050968	−0.10665
SCOOHPI10	0.527494	0.539186	0.101808	−0.18828	−0.03109	−0.13691
SCOOHPI11	0.617869	0.475583	0.047775	−0.18815	0.049453	0.025043
SCOOHPI12	−0.09953	0.888057	−0.00419	0.043593	0.110158	−0.03919
SCOOHPI13	0.118379	0.573288	0.101746	0.218041	−0.18587	0.262853
SCOOHPI14	0.520805	0.333713	−0.00709	0.25562	−0.14991	0.293008
SCOOHPI15	0.927423	−0.18539	−0.02231	0.03629	0.134563	0.010929
SCOOHPI16	−0.08654	−0.02559	0.017144	0.018562	−0.05073	0.921207
SCOOHPI17	0.062192	−0.08554	0.777565	−0.30566	0.295394	0.184732
SCOOHPI18	0.088248	0.13756	0.019484	0.111856	0.871988	−0.07236

## Appendix C. Item Coordinates (Varimax Rotation)

	**Factor 1**	**Factor 2**	**Factor 3**	**Factor 6**	**Factor 4**	**Factor 5**
SCOOHPI1	0.05	0.23	0.51	0.56	−0.16	−0.17
SCOOHPI2	0.17	0.05	0.79	0.17	0.09	−0.07
SCOOHPI3	−0.01	0.04	0.79	0.18	−0.15	0.01
SCOOHPI4	0.21	0.12	0.16	0.76	0.30	0.14
SCOOHPI5	0.13	0.52	−0.19	−0.06	0.15	0.12
SCOOHPI6	−0.08	0.66	0.19	0.24	0.19	−0.05
SCOOHPI7	0.39	0.58	0.16	0.49	−0.07	0.05
SCOOHPI8	0.80	0.28	0.23	0.19	0.00	0.02
SCOOHPI9	0.84	−0.03	0.02	0.09	0.08	−0.13
SCOOHPI10	0.59	0.53	0.10	−0.02	0.02	−0.08
SCOOHPI11	0.67	0.52	0.03	−0.06	0.05	0.02
SCOOHPI12	0.08	0.86	0.07	0.09	0.04	−0.06
SCOOHPI13	0.17	0.59	0.14	0.39	−0.13	0.21
SCOOHPI14	0.50	0.40	0.10	0.37	−0.16	0.28
SCOOHPI15	0.86	−0.07	0.01	0.11	0.18	0.01
SCOOHPI16	−0.10	0.04	−0.02	0.06	−0.04	0.91
SCOOHPI17	0.14	0.01	0.62	−0.36	0.38	0.25
SCOOHPI18	0.16	0.19	−0.02	0.10	0.87	−0.08

## Appendix D. Polychoric Correlation Matrix Between Items of the SCOOHPI Scale

	**SCOOHPI1**	**SCOOHPI2**	**SCOOHPI3**	**SCOOHPI4**	**SCOOHPI5**	**SCOOHPI6**	**SCOOHPI7**	**SCOOHPI8**	**SCOOHPI9**
SCOOHPI1	1	0.458053	0.437338	0.384353	−0.01814	0.286295	0.469632	0.284403	0.111708
SCOOHPI2	0.458053	1	0.530406	0.286235	0.045792	0.246168	0.296086	0.411538	0.141408
SCOOHPI3	0.437338	0.530406	1	0.217718	−0.10464	0.202296	0.209447	0.205591	0.027281
SCOOHPI4	0.384353	0.286235	0.217718	1	0.111542	0.264539	0.501867	0.352078	0.193487
SCOOHPI5	−0.01814	0.045792	−0.10464	0.111542	1	0.178512	0.263948	0.253926	0.060674
SCOOHPI6	0.286295	0.246168	0.202296	0.264539	0.178512	1	0.434775	0.266577	0.097252
SCOOHPI7	0.469632	0.296086	0.209447	0.501867	0.263948	0.434775	1	0.639206	0.320734
SCOOHPI8	0.284403	0.411538	0.205591	0.352078	0.253926	0.266577	0.639206	1	0.69588
SCOOHPI9	0.111708	0.141408	0.027281	0.193487	0.060674	0.097252	0.320734	0.69588	1
SCOOHPI10	0.241102	0.157199	0.125075	0.236265	0.263979	0.231519	0.535408	0.537675	0.38967
SCOOHPI11	0.153356	0.133306	0.038029	0.240666	0.234154	0.252887	0.498231	0.631002	0.473636
SCOOHPI12	0.270038	0.110194	0.086182	0.236113	0.290991	0.593495	0.522109	0.315113	0.110163
SCOOHPI13	0.391448	0.145599	0.222702	0.411699	0.259066	0.348467	0.607615	0.365327	0.141593
SCOOHPI14	0.32464	0.205081	0.143849	0.439672	0.18996	0.239322	0.648342	0.565632	0.328399
SCOOHPI15	0.064473	0.201615	0.025858	0.290497	0.132551	0.043912	0.273051	0.634229	0.697753
SCOOHPI16	−0.04784	−0.06007	0.040526	0.04784	0.050383	0.093536	0.035834	−0.01633	−0.13634
SCOOHPI17	0.06575	0.339193	0.232796	0.094951	−0.0468	0.01043	0.025911	0.149633	0.093633
SCOOHPI18	0.030827	0.08456	−0.04943	0.251735	0.163742	0.216706	0.181138	0.19913	0.209024

	**SCOOHPI10**	**SCOOHPI11**	**SCOOHPI12**	**SCOOHPI13**	**SCOOHPI14**	**SCOOHPI15**	**SCOOHPI16**	**SCOOHPI17**	**SCOOHPI18**
SCOOHPI1	0.241102	0.153356	0.270038	0.391448	0.32464	0.064473	−0.04784	0.06575	0.030827
SCOOHPI2	0.157199	0.133306	0.110194	0.145599	0.205081	0.201615	−0.06007	0.339193	0.08456
SCOOHPI3	0.125075	0.038029	0.086182	0.222702	0.143849	0.025858	0.040526	0.232796	−0.04943
SCOOHPI4	0.236265	0.240666	0.236113	0.411699	0.439672	0.290497	0.04784	0.094951	0.251735
SCOOHPI5	0.263979	0.234154	0.290991	0.259066	0.18996	0.132551	0.050383	−0.0468	0.163742
SCOOHPI6	0.231519	0.252887	0.593495	0.348467	0.239322	0.043912	0.093536	0.01043	0.216706
SCOOHPI7	0.535408	0.498231	0.522109	0.607615	0.648342	0.273051	0.035834	0.025911	0.181138
SCOOHPI8	0.537675	0.631002	0.315113	0.365327	0.565632	0.634229	−0.01633	0.149633	0.19913
SCOOHPI9	0.38967	0.473636	0.110163	0.141593	0.328399	0.697753	−0.13634	0.093633	0.209024
SCOOHPI10	1	0.670175	0.392076	0.46258	0.422065	0.459205	−0.09253	0.136085	0.263264
SCOOHPI11	0.670175	1	0.500048	0.336302	0.496743	0.497802	0.00486	0.120193	0.267242
SCOOHPI12	0.392076	0.500048	1	0.453876	0.453292	0.068566	0.000171	0.054788	0.197335
SCOOHPI13	0.46258	0.336302	0.453876	1	0.453923	0.126969	0.147469	0.063857	0.076386
SCOOHPI14	0.422065	0.496743	0.453292	0.453923	1	0.371505	0.146824	0.093406	0.060034
SCOOHPI15	0.459205	0.497802	0.068566	0.126969	0.371505	1	−0.04844	0.105074	0.25576
SCOOHPI16	−0.09253	0.00486	0.000171	0.147469	0.146824	−0.04844	1	0.025155	−0.05653
SCOOHPI17	0.136085	0.120193	0.054788	0.063857	0.093406	0.105074	0.025155	1	0.197108
SCOOHPI18	0.263264	0.267242	0.197335	0.076386	0.060034	0.25576	−0.05653	0.197108	1
